# Editorial: Transient receptor potential (TRP) ion channels in non-excitable cells

**DOI:** 10.3389/fphys.2023.1213332

**Published:** 2023-05-16

**Authors:** Mustafa Nazıroğlu, Beatrice Mihaela Radu, Dana Cucu

**Affiliations:** ^1^ Department of Biophysics, Medical Faculty, Suleyman Demirel University, Isparta, Türkiye; ^2^ Neuroscience Research Center, Suleyman Demirel University, Isparta, Türkiye; ^3^ Department of Anatomy, Animal Physiology and Biophysics, Faculty of Biology, University of Bucharest, Bucharest, Romania

**Keywords:** non-excitable neurons, TRP channels, fibrosis, neuronal disease, squamous cell carcinoma

Non-excitable cells have no voltage-gated sodium or calcium channels and are unable to generate action potentials in response to depolarizing stimuli. As a result, changes in membrane potential primarily affect Ca^2+^ influx via various channel families. In the non-excitable cells, there is no voltage-gated calcium or sodium channels, and they are characterized by an inability to generate all-or-none action potentials in response to depolarizing stimuli. As a result, it is believed that the changes in membrane potential primarily affect intracellular free Ca^2+^ [(Ca^2+^)_i_] responses by changing the force that drives Ca^2+^ influx via ligand (chemical)-gated or second messenger-operated channels. There are well-known Ca^2+^ permeable cation channels such as ligand (chemical)-gated or second messenger-operated channels in the non-excitable cells, and they are responsible for the induction of membrane potential. One of them, the transient receptor (TRP) channel superfamily, was discovered during the last decades.

The TRP superfamily is responsible in the non-excitable cells for the inductions of several physiological functions such as phagocytosis and microglia activation. The TRP superfamily contains 28 members in the mammalian cells and is sub-divided into seven major subgroups such as canonical (TRPC), no mechanoreceptor potential (TRPN or NOMPC), vanilloid (TRPV), ankyrin (TRPA), melastatin (TRPM), polycystin (TRPP), and mucolipin (TRPML). The TRP proteins are mostly unselective channels for monovalent and divalent cations that are activated by various stimuli, including heat, cold, chemical substances, oxidative stress, mechanical, and osmotic stress. In the last decade, the report numbers of TRP channels were remarkably increased in the literature. In 2021, David Julius was Nobel prize awarded for the molecular identification of TRP channels. Understanding the TRP channel tissue expression, activation, and inhibition mechanisms added great progress on the importance of TRP channels in cardiac disorders, diabetes, neurodegenerative diseases, and cancer. We know that the TRPV1 channels modulate neuropathic pain and body temperature changes, whereas the TRPM subfamily participates in the migration and proliferation of several cancer types such as prostate and glioblastoma. In addition, limited recent data indicated that the TRP channels involve in the action of non-excitable cells.

Together, the publications that have been published on the current Research Topic contribute to our understanding of the role that TRP channels play in the stimulation of non-excitable cells during cardiac function and cancer, as well as the identification of novel prospective therapeutic targets.

Hypertensive nephropathy is a major cause of end-stage renal disease. Accumulating data indicate that moderate exercise decreases blood pressure and delays renal fibrosis, although the impact of different exercise training and the underlying mechanism on renal function have not been fully clarified yet. Zhao et al. provide an original paper that investigates whether high-intensity exercise training (HIET) could exacerbate renal inflammatory conditions and activate TRPV4-TGF-1-Smad2/3-CTGF-mediated renal fibrotic pathways in hypertension, resulting renal damage and fibrosis. The authors used rats and normal rat kidney interstitial fibroblast cells (NRK-49F) and performed Western immunoblotting, immunohistochemical, and lactate assays in the animal tissue and cell culture samples. In addition, the protein levels of TRPV4 were also investigated in the renal cortex of rats and NRK-49F cells. The authors found the concentration of lactic acid to be correlated with expression levels of TGFβ-1 and TRPV4 as key signaling molecules in fibrosis formation. Overall, Zhao et al. confirm that inhibiting the renal fibrosis in the hypertension phenotypes is accomplished by reducing the TRPV4 and lactate.

Numerous neurological disorders, including neurodegenerative diseases, glaucoma, epilepsy, and myalgic encephalomyelitis/chronic fatigue syndrome, are linked to mutations and protein misfolding in the membrane proteins of cation channels. Myalgic encephalomyelitis/chronic fatigue syndrome and other neurological illnesses are significantly impacted by the TRPM3 cation channel mutations. The primary transcript of the hTRPM3 membrane protein encompasses 1,555 amino acids and the TRP signature motif (XWKFXR). To help discovering the TRPM3 proteotypic peptides for further identification of isoforms in the natural killer cells of patients and control subjects, Magawa et al. optimized a detergent-based protein extraction procedure of TRPM3 channel from nature killer cells. This protocol now informs future functional and structural proteomic research, and extends o TRPM3 studies to the natural killer cells of the patients.

Fibrotic diseases have high morbidity and mortality. TRPV1 channel has recently linked to fibrosis and fibrotic organs, including the kidney, lung, heart, and cornea. Peng et al. reviewed and analyzed the correlation between the TRPV1 channel and fibrotic diseases. The authors highlighted the importance of TRPV1 for the induction of fibrosis through TRPV1 stimulation-induced inflammation, oxidative stress, the renin angiotensin system, and transforming growth factor beta (TGF- β)/SMAD signaling. Finally, they demonstrated that the TRPV1 stimulation-induced Ca^2+^ influx, oxidative stress, neuropeptides, and inflammation collectively cause the induction of fibrosis in several diseases. However, they claimed that additional studies are required to confirm the inhibition of the TRPV1 channel for the treatment of fibrotic diseases.

In the fourth paper, Gwanyanya and Mubagwa reviewed involvement of TRP cation channels in the cardiac fibroblast pathophysiology. Cardiac fibrosis was induced via the excessive activation of fibroblasts by the various mechanical and chemical signaling factors, including the inflammation-induced stress factors, temperature changes, neurohumoral factors (such as aldosterone and angiotensin II), and the TGF-β1. In turn, the factors induce NADPH oxidases-mediated reactive free oxygen species (ROS) productions and excessive Ca^2+^ influx. Hence, the excessive Ca^2+^ influx plays an important role in fibroblast action and myofibroblast contraction as well as trigger oxidative stress involved in pathological cardiac fibrosis. Several TRP channels such as TRPA1, TRPV1, and TRPV4 are activated by the factors. There is also a direct relationship between the TRP channel activation and the fibrosis etiology. The authors remarked involvement of TRPC (TRPC3 and TRPC5), TRPM (TRPM2, TRPM4, and TRPM7), TRPV (TRPV1 and TRPV3), TRPA1, and TRPP channels in the etiology of fibrosis. In the paper, the interaction between fibroblast TRPs and chronic myocardial metal toxicity was also evaluated. They demonstrated that the physiologic balance between the profibrotic and antifibrotic factors was arranged in fibroblasts by the TRP channels-stimulation mediated Ca^2+^ influx.

The original paper of Sun et al. described how capsazepine (CPZ) analog CIDD-99 decreased proliferation of the squamous cell carcinoma (SCC). The incidence of SCC is enormously increasing around the world, and its etiology is not sufficiently understood. CPZ is an antagonist of TRPV1 channels. Sun et al. produced CIDD-99, and tested it in the human OSCC cell lines, Ca-27 and HSC-3. Using the cell viability, Western blotting, Calcium imaging (Fura-2), and patch-clamp analyses, the authors demonstrated that CIDD-99 inhibited TRPC1, decreasing endoplasmic reticulum calcium levels via inducing the endoplasmic reticulum stress. The different doses of CIDD-99 also decreased STIM1 expression levels. The authors suggested that CIDD-99 stimulates apoptosis via an endoplasmic reticulum stress-induced cell death pathway and TRPC1 channel inhibition.

This Research Topic provides an overview about the multiple consequence stimulation or inhibition of TRPM3, TRPV1, and TRPV4 channels in the pathogenesis of fibrosis and neurological diseases. The articles in this Research Topic provide an abstract of the multiple roles of non-excitable cells via the stimulation or inhibition of TRPM3, TRPV1, and TRPV4 channels in the pathogenesis of fibrosis and neurological diseases, and provide emerging evidence as a target for the development of therapeutic agents to modulate pathological states and improve neurological and fibrotic abnormalities.

